# Intercomparison of In Situ Sensors for Ground-Based Land Surface Temperature Measurements

**DOI:** 10.3390/s20185268

**Published:** 2020-09-15

**Authors:** Praveena Krishnan, Tilden P. Meyers, Simon J. Hook, Mark Heuer, David Senn, Edward J. Dumas

**Affiliations:** 1 NOAA ARL Atmospheric Turbulence and Diffusion Division, Oak Ridge, TN 37830, USA; tilden.meyers@noaa.gov (T.P.M.); mark.heuer@noaa.gov (M.H.); d.l.senn@noaa.gov (D.S.); ed.dumas@noaa.gov (E.J.D.); 2 Oak Ridge Associated Universities, Oak Ridge, TN 37830, USA; 3 Jet Propulsion Laboratory California Institute of Technology, Pasadena, CA 91109, USA; Simon.J.Hook@jpl.nasa.gov

**Keywords:** land surface temperature, infrared temperature sensors, thermal imaging

## Abstract

Land surface temperature (LST) is a key variable in the determination of land surface energy exchange processes from local to global scales. Accurate ground measurements of LST are necessary for a number of applications including validation of satellite LST products or improvement of both climate and numerical weather prediction models. With the objective of assessing the quality of in situ measurements of LST and to evaluate the quantitative uncertainties in the ground-based LST measurements, intensive field experiments were conducted at NOAA’s Air Resources Laboratory (ARL)’s Atmospheric Turbulence and Diffusion Division (ATDD) in Oak Ridge, Tennessee, USA, from October 2015 to January 2016. The results of the comparison of LSTs retrieved by three narrow angle broadband infrared temperature sensors (IRT), hemispherical longwave radiation (LWR) measurements by pyrgeometers, forward looking infrared camera with direct LSTs by multiple thermocouples (TC), and near surface air temperature (AT) are presented here. The brightness temperature (BT) measurements by the IRTs agreed well with a bias of <0.23 °C, and root mean square error (RMSE) of <0.36 °C. The daytime LST(TC) and LST(IRT) showed better agreement (bias = 0.26 °C and RMSE = 0.67 °C) than with LST(LWR) (bias > 1.1 and RMSE > 1.46 °C). In contrast, the difference between nighttime LSTs by IRTs, TCs, and LWR were <0.47 °C, whereas nighttime AT explained >81% of the variance in LST(IRT) with a bias of 2.64 °C and RMSE of 3.6 °C. To evaluate the annual and seasonal differences in LST(IRT), LST(LWR) and AT, the analysis was extended to four grassland sites in the USA. For the annual dataset of LST, the bias between LST (IRT) and LST (LWR) was <0.7 °C, except at the semiarid grassland (1.5 °C), whereas the absolute bias between AT and LST at the four sites were <2 °C. The monthly difference between LST (IRT) and LST (LWR) (or AT) reached up to 2 °C (5 °C), whereas half-hourly differences between LSTs and AT were several degrees in magnitude depending on the site characteristics, time of the day and the season.

## 1. Introduction

Land surface temperature (LST), the thermodynamic temperature of the interface between the Earth’s surface and its atmosphere, is a key variable in the determination of land surface–atmosphere processes from local to global scales. LST, also referred to as skin temperature of land surface, has been identified as one of the most important environmental data records [1] and is widely used in meteorological, climatological, hydrological, ecological, biophysical, and biochemical research [2,3,4,5,6,7]. The magnitude and temporal variation of LST is determined by the conductive, convective, and radiative energy exchange process at the earth atmosphere interface in response to the solar insolation and surface characteristics. LST, usually measured by ground-based, airborne, and space-borne remote sensing instruments, is the aggregated radiometric temperature of all surface components including soil, vegetation, and other land surface components within the sensor field of view in the direction of observation [8,9]. The measurement of LST by radiometric sensors, indicative of the thermal state of the surface, differs from near surface air temperature (AT) routinely measured at weather stations using a sheltered thermometer 1.5–3.5 m above a flat, grassy, well-ventilated surface or ground-surface temperature, usually measured by thermistors up to 5 cm beneath the surface cover [10]. As satellites do not directly measure AT, the satellite infrared-based LST measurements have been widely used for the indirect estimation of AT [11,12,13,14].

Accurate measurements of LST at high spatial and temporal resolutions are needed to improve the model parameterizations of land-atmosphere exchange processes and for assessing the uncertainty in both climate and numerical weather prediction models [15,16,17]. With advances in remote sensing, satellite-based LST can be estimated globally by the inversion of Planck’s law from the top of the atmosphere radiances in the thermal infrared (IR) and microwave (MW) atmospheric windows as the total radiative energy emitted by the surface is a function of temperature. Of these, clear-sky LST retrieval from IR is widely used due to its stronger dependence of radiation on temperature, better accuracy, high spatial resolution, and smaller variation of surface emissivity in these wavelengths, when compared to all-sky MW LSTs with coarse spatial resolution, low temperature retrieval accuracy, and shortage of long-term MW dataset [18,19]. In the thermal infrared spectral atmospheric window region (8–14 μm), LST is typically retrieved by the surface emitted radiance received at the sensor for a given wavelength, after considering the atmospheric attenuation effects, as a function of both the actual surface temperature and emissivity. The methods and algorithms for the retrieval of satellite LST, based on radiative transfer models [20,21], are going through considerable evolution, but a consensus of globally applicable algorithm for long-term datasets from different platforms has not yet been achieved [7,22]. Even though satellite radiometric measurements of LST are a powerful tool, there are still large uncertainties associated with the retrieval of remotely sensed LST measurements. To improve confidence in the methods, algorithms, and parameters used to derive remotely sensed LST, and to assess accuracy and precision of the retrieved LST, validation of satellite-based LST is required. One of the most popular and accurate methods for satellite LST validation is referred to as temperature based (T-based) method which is the direct comparison of satellite LST with ground-based in situ measurements using thermal infrared radiometers over thermally homogeneous field sites concurrently with satellite overpass [7,19,23]. Even though satellite-based LST provides spatial variation, its temporal variation is limited when compared to ground-based measurements of LST. This demands more accurate ground measurements of LST for longer periods over various land covers to fill spatial and temporal gaps of current satellite measurements. However, such measurements are quite scarce.

As the surface skin layer is in contact with both atmosphere and soil/vegetation, it is difficult to measure LST using traditional thermometers. The in situ LST is not directly measured, but estimated from surface brightness temperature (BT) derived using the amount of energy radiated by the surface, target emissivity and sky radiance derived from incoming longwave radiation or sky BT measurements to account for the upward reflected component of the downward radiation. Currently, there are three methods to obtain ground-based LST including the use of narrow angle broadband or multiband infrared radiometers or imagers, estimates from broadband infrared hemispheric fluxes, and using AT as a proxy for LST [24,25,26,27]. Contrary to the near surface AT, accurate ground-based LST measurements are not usually conducted globally as part of standard meteorological observations on a global basis. However, a few exceptions in the USA include BT measurements by narrow-angle IR thermometers (IRT) at the United States Climate Reference Network (USCRN, https://www.ncdc.noaa.gov/crn/) [28] and LST data obtained from broadband infrared hemispheric fluxes measured by pyrgeometers at the Surface Radiation Network (SURFRAD, https://www.esrl.noaa.gov/gmd/grad/surfrad/) [29,30] mainly over grass surfaces. A similar method can be used to estimate LST over various land cover types using pyrgeometers at energy flux sites like FLUXNET (https://fluxnet.ornl.gov), where radiation measurements are usually available. Recent technical advancements led to the production of light weight forward looking infrared (FLIR) sensors in addition to low cost IR sensors. These FLIR cameras onboard aircraft or unmanned aerial vehicles (UAVs) can provide LST images over a larger area and are especially useful in campaign mode experiments compared to point measurements by IR sensors. The footprints of tower-based measurements are much smaller than those from infrared imagers or sensors on UAVs, aircraft or satellite-based sensors. However, it remains an evolving technique with limited resolution, accuracy, poor contrast, and low signal to noise ratios that needs to be fine-tuned to obtain higher accuracy of IRT’s. Even though all the IR sensors and imagers are factory calibrated, neither multiple sensors with a different field of view nor their field deployment, have been compared over a long duration.

One of the most challenging aspects of these intercomparisons in the field is the difficulty to find naturally homogeneous sites compared to well-controlled laboratory-based comparisons. Recently, a few attempts have been made to assess the uncertainties in situ LST under laboratory and field conditions during fiducial reference measurements for validation of surface temperature from Satellites (FRM4STS) experiment in 2016 [31,32] and field inter-comparison experiment (FICE) in 2017 [33]. However, these experiments utilized only directional narrow angle IR radiometers for a short duration. In our study, intercomparison of ground-based LST measurements were carried out using the three methods as mentioned above during intensive field campaign and also over multiple field sites for a year. In an effort to evaluate and better quantify the uncertainties in ground-based LST measurements, we conducted an intercomparison of LSTs using in situ sensors over an asphalt surface in a parking lot in Oak Ridge and extended the analysis on the methods of LST estimation to four grassland sites. The objectives of the present paper are (1) to compare the LST measurements made over an asphalt surface using point measurements by an array of thermocouples, three narrow angle IR thermometers, one set of pyrgeometers with a nearly hemispheric field of view, and a FLIR camera; (2) to assess how near-surface air temperature measurements made at the site compare with the ground-based LST measurements; and (3) to evaluate the difference in LST estimates using IRT and longwave radiation measurements at four grassland sites and compare it with near surface air temperature at those sites.

## 2. Materials and Methods

### 2.1. Sites and Measurements

The surface temperature measurements using multiple sensors used in this study were conducted at NOAA/ARL/ATDD, Oak Ridge, TN, USA (36.003576 N, 85.248738 W, elevation 259 m), during 10 October 2015 to 8 January 2016. The instruments were installed at 1.7 m at the middle of a ~5 m long horizontal truss mounted east-west over two tripods placed almost diagonally over the study area in the parking lot. The asphalt pavement (~6 m in diameter) was coated with asphalt emulsion driveway sealer for this experiment. The precipitation measurements are from a tipping bucket rain gauge (Model TB-3) from a co-located meteorological test station, within ~90 m from the site on the ATDD campus.

The surface temperatures used in the study were measured by three infrared radiometers—Apogee Infrared Temperature (IR) Sensors (SI-111 Infrared Radiometer, Apogee Instruments Inc., Logan, UT, USA), Heitronics IR radiometer (KT19.85 II, Heitronics, Infrarot Messtechnik GmbH, Wiesbaden, Germany), Jet Propulsion Laboratory’s Quasi Nulling IR Radiometer (here after JPLR) (500 series), one infrared imager—Forward Looking Infrared Radiometer (FLIR) Tau 2 camera (FLIR Systems, Inc., Wilsonville, OR, USA), and 12 thermocouples embedded on the asphalt surface. The main specifications of the IR radiometers are presented in Table 1. In addition to this, measurements of air temperature (Thermometrics corp PRT, Northridge, CA, USA), shortwave radiation (paired pyranometers, model CNR1-CM3, Kipp and Zonen, Delft, The Netherlands), and longwave radiation (paired pyrgeometers, CNR1-CG3, Kipp and Zonen, Delft, The Netherlands) were also made. Platinum resistance thermometer (PRT) was enclosed within a fan aspirated radiation shield to minimize radiative errors on air temperature. The CG3 is a hemispherical pyrgeometer with a nominal spectral range of 4.5–42.0 μm, operational temperature range of −40 to 80 °C, expected accuracy of ±10% for daily sums and a field of view (FOV) 150° (http://kippzonen.com). The effective radius of the FOV of pyrgeometers on a 1.7 m tower is ~6.34 m.

Heitronics KT19.85 II model IR Pyrometer (KT19.85 II, Heitronics, Infrarot Messtechnik GmbH, Wiesbaden, Germany) has a 1.5° half angle and measures surface temperatures with an accuracy of ±0.5 °C and has a temperature resolution of 0.03 °C. The spectral sensitivity is between 9.6 and 11.5 µm [34]. The footprint of the Heitronics IR Pyrometer varies as a function of the altitude (i.e., when the sensor was at 1.7 m above the ground, the footprint of the sensor at the ground was a circle with a diameter of 0.089 m and area of ~0.0062 m^2^).

Apogee infrared radiometers (Apogee model SI-111, Apogee Instruments Inc., Logan, UT, USA) have a 22° half angle FOV and detect radiation in the 8–14 µm wavelength range. It has a stated absolute accuracy of ±0.5 °C from −40 to 70 °C and ±0.2 °C from −10 to 65 °C. (www.apogeeinstruments.com/). At 1.7 m, the footprint of the sensor at the ground was a circle with 1.37 m in diameter.

JPL Quasi Nulling IR Radiometer (500 series) (here after JPLR) is an autonomous, self-calibrating, field portable radiometer developed at JPL and calibrated to work in the range from 4 to 40 °C with an accuracy of ±0.1 °C. It has a half-angle FOV of 18° and works in the 8–14 µm range. This sensor was originally designed to measure surface temperature of water bodies similar to JPL near-nulling radiometer (http://calval.jpl.nasa.gov/radiometers). The footprint at 1.7 m for the sensor was a circle with ~1.1 m in diameter [35].

The thermal infrared imager used in this study was a Forward-Looking Infrared Radiometer (FLIR) Tau 2 model camera (FLIR Systems, Inc., Wilsonville, OR, USA, www.flir.com) with a 336 × 256 pixel image dimension and a 7.5 mm lens. The thermal camera used an Uncooled VOx Microbolometer to detect longwave radiation between 7.5 and 13.0 μm. The camera operates at ambient temperature of −40 °C to +80 °C and measures scene temperature within the range of −40 °C to +165 °C. The lens FOV is 45° × 35°, so at 1.7 m it captures images with an area of 1.41 × 1.07 m^2^. The camera was controlled by a TeAx Thermal Capture data acquisition system (TeAx, Wilnsdorf, Germany) and can store data at 7.5 Hz. This imager has accuracy on the order of ±5 °C or 5% in high-gain state with advanced radiometry features and can vary slightly across the full operating temperature range [36] (www.flir.com).

To measure the actual LST, 12 thermocouples were embedded on the surface in the footprint of the IR instruments. The type K (Nickel-Chromium/Nickel-Alumel) thermocouples have an accuracy of 0.75% or (2.2 °C) and works in the temperature from −200 °C to 1260 °C (http://www.thermometricscorp.com/thertypk.html). We used the LST measurement of eight thermocouples which behaved closely during the experiment. The cables were also embedded on the ground and covered with asphalt emulsion coating. All IR sensors were mounted at the center of the horizontal truss and oriented to look straight down. All the measurements, except FLIR and TC were sampled every 30 s and averaged to 5 min (using data-logger, model CR23X, Campbell Scientific Inc., Logan, UT, USA). Thermocouple measurements were sampled at 2 s and averaged to 5 min using another data logger. FLIR measurements were conducted at random intervals during the daytime of DOY 341 to 344 in 2015. Each time, 100 images were collected and were averaged to get mean surface temperature.

**Figure 1:**
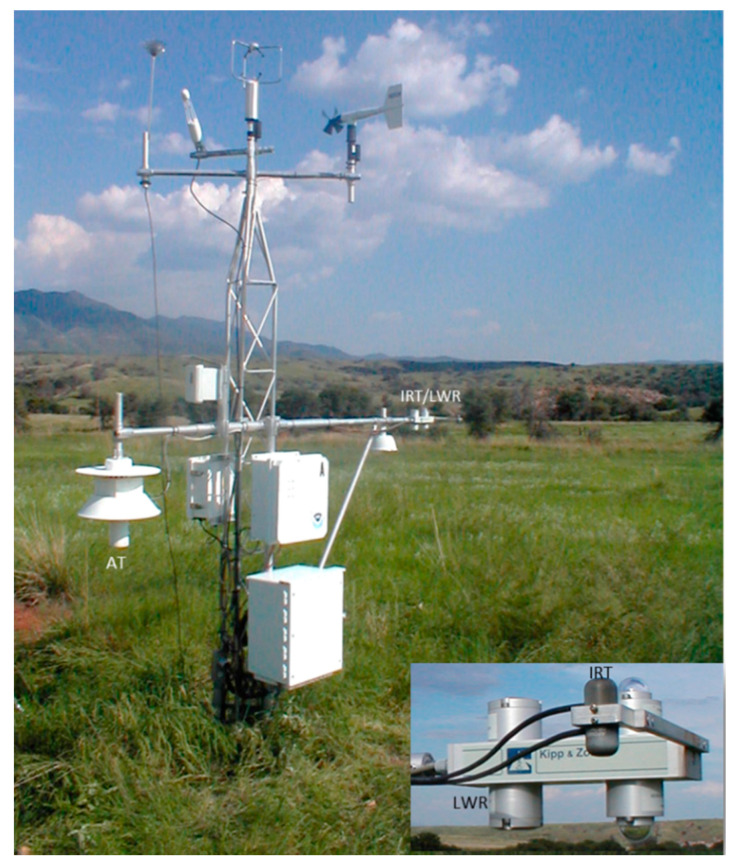
Picture of the Surface Energy Budget Network (SEBN) site located in Audubon, AZ. The insert shows Apogee IRTS-P (IRT) and Kipp and Zonen (CNR1-CG3) (LWR) sensors.

To extend the data analysis and to evaluate the results on the methods of LST measurements, we have used half-hourly data from four grassland sites (Audubon, Brookings, Canaan Valley, and Fort Peck) (Table 2) with measurement heights below 3 m to represent traditional near surface AT that has been used as proxy for LST in many studies. The CNR and IRT were mounted on a ~2 m boom, but close to each other so that the footprints overlap each other (Figure 1) These sites were established for NOAA’s Surface Energy Budget Network (SEBN) (https://www.atdd.noaa.gov/sebn/) and are also part of the Ameriflux network (https://ameriflux.lbl.gov/).

The surface temperatures from these sites were measured using Apogee IRTS-P Infrared Temperature Sensor (Model IRTS-P; Apogee Instruments Inc., Logan, UT, USA), an older version of the Apogee IRT sensor used in the study above. The sensor has an accuracy of ±0.3 °C from −10 to 55 °C. This highly water-resistant sensor used two type-K shielded thermocouple outputs, one for target and one for sensor body temperature which is used for corrections of target temperature. The spectral range of the sensor is from 6.5 to 14 μm and has a halfangle FOV of 28°. The radiation measurements were performed by a CNR1 net radiometer (Kipp and Zonen) and air temperature using platinum resistance thermometers (Thermometrics Corp PRT, Northridge, CA, USA). Precipitation was measured with a weighing rain gauge at all sites (Hydrol. Serv.). One year of data from all sites are selected for this analysis. In addition to this, the data from co-located SURFRAD (48.31 N, 105.10 W) and USCRN (Wolf Point 29 ENE, 48.30 N, 105.10 W) sites at Fort Peck were also included in the analysis. The AT and BT (available only from DOY 175 in 2012) measurements at the USCRN site were performed by using platinum resistance thermometers mentioned above and Apogee Infrared Temperature (IRT) Sensors (SI-311 Infrared Radiometer, Apogee Instruments Inc., Logan, UT, USA, spectral range 6.5–14 µm, 28° half-angle FOV, with an accuracy of ±0.2 °C) at 1.25 m, respectively. At the SURFRAD site, the upwelling and downwelling thermal infrared irradiances were measured by two pyrgeometers at 10 m level (Eppley Precision Infrared Radiometer with spectral range 3.5 to 50.0 µm, FOV of 180° and an accuracy of 4.2 Wm^−2^) and air temperature was measured by using a precision resistance thermistor with an accuracy of ±0.5 °C.

### 2.2. Data Analysis

#### 2.2.1. Estimation of LST from Brightness Temperatures

The total radiation received by an infrared sensor or camera is a combination of the radiation emitted by the target surface and the reflected radiation from the surroundings. This radiant energy can be attenuated, absorbed, and reemitted by the atmosphere between the surface and the sensor before reaching the sensor. The contributions from the atmosphere are mainly determined by the transmittance of the atmosphere (τ) and emittance of the atmosphere (1 − τ). Here the value of τ is influenced by the temperature, the relative humidity, and the distance between the sensor and target surface [20,37,38,39,40]. As the sensors are mounted very close to the surface in this study the atmospheric contribution is negligible (τ~1). Therefore, radiant energy detected by the sensor (*L_Sensor_*) can be expressed as

(1) $$L_{Sensor}=\varepsilon L_{Target}+\left(1-\varepsilon \right)L_{↓}$$

where *L_Target_* is the radiant energy emitted by the target surface, *ε* is the surface emissivity, *L*_↓_ is the incoming atmospheric radiation at the surface, and 1 − *ε* corresponds to the reflectivity. *L_Sensor_* = *B*(*T_b_*), *L_Target_* = *B*(*T_s_*) and *L*_↓_ = *B*(*T_sky_*), where *T_b_* is the brightness temperature known as equivalent blackbody temperature [8], *T_s_* is the surface radiometric temperature or LST (in K), *T_sky_* is the background or sky brightness temperature, and *B* is the Planck function integrated over a wavelength band for a given the spectral emissivity. Ideally, for a blackbody, *B*(*T*) can be calculated by integrating the Planck function over the entire spectral region, resulting in the Stefan–Boltzmann law [41,42], so that $L_{Sensor}=\sigma T_{b}^{4}$, $L_{Target}=\sigma T_{s}^{4}$, and $L_{↓}=\sigma T_{sky}^{4}$ [43] where *σ* is the Stefan–Boltzmann constant (5.67 × 10^−8^ Wm^−2^ K^−4^). This is a reasonable approximation for the measured directional surface radiances in the 8–14 μm wavelength bands for a limited range of temperatures [9]. Based on Equation (1), *T_s_* by IRTs were obtained as

(2) $$T_{s}={\left(\frac{\sigma T_{b}^{4}-\left(1-\varepsilon \right)L_{↓}}{\varepsilon \sigma }\right)}^{\frac{1}{4}}$$

The upwelling and downwelling broadband hemispherical radiances measured by the pyrgeometers were used to estimated ground-based *T_s_* as

(3) $$T_{s}={\left(\frac{L_{↑}-\left(1-\varepsilon \right)L_{↓}}{\varepsilon \sigma }\right)}^{\frac{1}{4}}$$

In this study, to correct the IRT brightness temperatures, Equation (2) was used with *L*_↓_ measurements by CNR1 pyrgeometers instead of using sky temperature measurements to estimate incoming radiation as the method to receive *L*_↓_ has negligible effect on the estimation of *T_s_* [44]. In our study the emissivity settings of all IR sensors and cameras were set as 1 so they provided measurement of *T_b_* while the thermocouples embedded on the surface measured the actual *T_s_*. The estimated values of LST using *T_b_* measurements by IRTs, and those by longwave radiation measurements, are referred to as the *T_s_* (IRT) and *T_s_* (LWR), respectively, in the subsequent sections, while the direct LST measurement by thermocouples are denoted by *T_s_* (TC).

#### 2.2.2. Surface Emissivity

The broad band emissivity of any surface depends on the material type, surface characteristics, composition, roughness, soil moisture, angle, and direction of emission, wavelength, or spectral of infrared [45,46]. The direct method to estimate surface emissivity in the laboratory experiment involves the comparison of the radiant temperature between the measured samples and blackbody. However, direct measurement of surface emissivity in the field is practically difficult due to cost and instrumental system [31,46,47,48,49,50]. For known values of the *T_s_* (here by thermocouple) and *T_b_* (by IRT), the surface emissivity can be estimated using Equation (2) as

(4) $$\varepsilon =\left(\frac{\sigma T_{b}^{4}-L_{↓}}{\sigma T_{s}^{4}-L_{↓}}\right)$$

In this study, a simple method was to estimate *ε*, as the slope of the regression through origin with $\sigma T_{b}^{4}-L_{↓}$ against $\sigma T_{s}^{4}-L_{↓}$ for all data for the study period. It was found to be 0.902 ± 0.0002 (S.E), the emissivity of the surface. To evaluate this result, we carried out a regression analysis with measured LST using thermocouple with those estimated by using Equation (2) with emissivity from 0.65 to 1, in steps of 0.001. The absolute bias and RMSE in LSTs are estimated for every value of *ε*. The absolute bias between direct measurement by thermocouple and estimated LST was lowest (0.0062 °C) for a value 0.90 agreeing with our estimation of *ε* of the target surface. This value falls in the lower end of the range of values (0.9–0.98) reported for surface containing asphalt. So, we used *ε* as 0.90 for correcting the measured temperature by the IR sensors and camera using Equation (2) for the experiment conducted over the parking lot. However, for the field sites we have used the reported value of MODIS-based *ε* for the pixel containing the tower locations due to lack of in situ surface emissivity measurements at the sites. They are 0.975, 0.987, 0.987, and 0.987 for Audubon, Brookings, Canaan Valley, and Fort Peck grasslands, respectively [51].

In this study, daytime clear-sky condition refers to periods with solar radiation >10 Wm^−2^ and clearness index (CI) >0.70. Here the CI is the ratio of global radiation measured at the campaign site to the theoretical global radiation received on a horizontal surface placed at the top of the atmosphere (TOA) and it was calculated at every time step using the solar constant, day number, the latitude of the location, the solar declination angle, and the hour angle as described in [52]. To compare the surface temperature measurements made by the sensors, linear regression analysis is performed and the coefficient of determination (*r*) was estimated. The mean bias, standard deviation of the difference (STDd) and root mean square error (RMSE) were estimated using ∆*T* = *y* − *x*, where *y* and *x* are the independent and dependent variables, respectively. The mean bias, STDd, and RMSE are used as the measure of accuracy, precision, and uncertainty, respectively [53]. In addition to this, linear regression analysis was performed and correlation coefficients were estimated.

## 3. Results

### 3.1. Comparison of Surface Temperature Measurement Using IRTs, FLIR Camera, and Thermocouples

**Figure 2:**
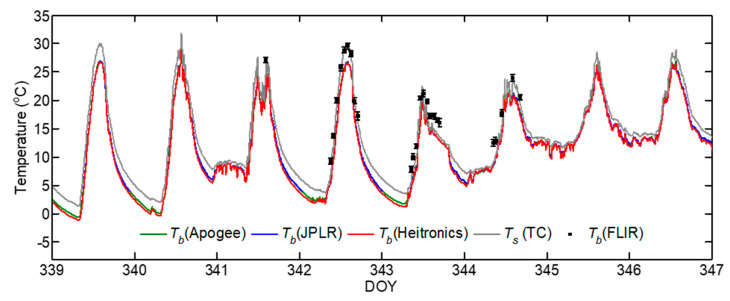
Comparison of 5-min surface brightness temperature (*T_b_*) measurement during DOY 339 to 347 in 2015 using Apogee, Heitronics, and JPLR. For JPLR, the temperature measurements within calibation range from 4 to 40 °C only are shown. The mean LST (*T_s_*) measured by thermocouples (TC) are shown by gray lines. The average and standard deviation of the forward looking infrared (FLIR) *T_b_* measurements are shown by black squares and vertical bars, respectively.

**Figure 3:**
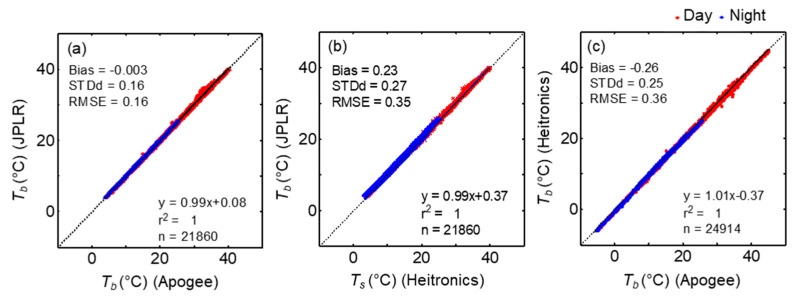
Comparison of 5-minute surface brightness temperature measurements (*T_b_*) by IRT sensors—(**a**) JPLR vs. Apogee, (**b**) JPLR vs. Heitronics, and (**c**) Heitronics vs. Apogee during the campaign period. Mean bias, STDd, RMSE, and results of the linear regression analysis (solid line) for the whole dataset are shown. The dotted line indicates 1:1 relationship between the variables.

To examine how the surface temperature measurement by multiple IRTs compare with the direct measurement of *T_s_* by thermocouple, the time series of the brightness temperature (*T_b_*) measurements made at the parking lot during a selected period DOY 339–347 containing a period with FLIR measurements are shown in Figure 2. Here, *T_b_* measurements by IRTs and FLIR, rather than LST were used to minimize any biases associated with the choice of surface emissivity and the correction for reflectivity effects on *T_b_*. The time series of surface temperature measurements by all sensors captured the diurnal variations very well. The magnitude and changes in *T_b_* by IRTs showed strong agreement, with exceptions mainly during the nighttime. The temporal variation of *T_b_* and *T_s_* measurements indicate that *T_b_* was always lower than *T_s_* and the difference can be >3 °C at midday during clear air conditions. This shows the effect of emissivity correction (Section 2.2) on the magnitude of surface temperature measurements by IRTs at unit emissivity. The difference in *ε* alone can result in higher magnitudes of *T_s_* than *T_b_* even for similar *L*_↓_ in Equation (2) [44]. However, *T_b_* measurements by FLIR were higher than those by IRTs especially during clear sky conditions on DOY 342. The relationship between *T_b_* measurements by IRTs reveal a highly significant linear relationship with *r^2^*~1 (Figure 3).

For the entire dataset, the mean bias between *T_b_* by JPLR and Apogee were smaller than those between Heitronics and Apogee sensors or JPLR and Heitronics (Table 3). These biases are within the accuracy of the sensors (see Section 2). The linear regression relationship showed better results between JPLR and Apogee BTs when compared to the relationship with Heitronics and JPLR BTs. This is largely attributed to the similar footprint of both sensors (>1 m in diameter) compared to the relatively small footprint of Heitronics (0.089 m diameter). These areas partially overlap with the footprint of the FLIR camera, a rectangle with an area of 1.41 × 1.07 m^2^.

**Figure 4:**
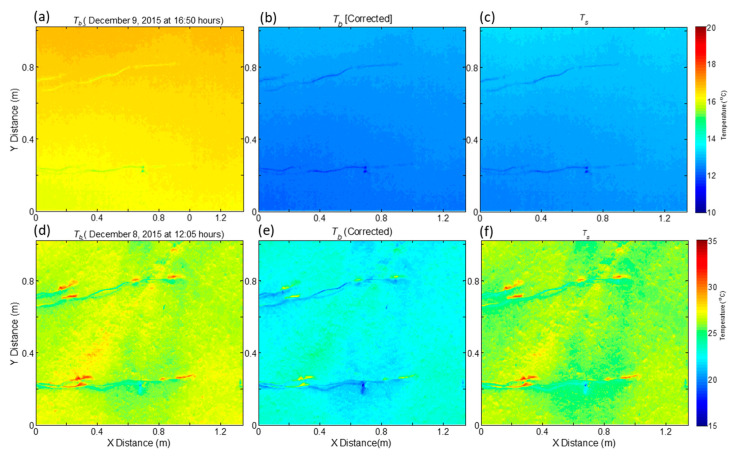
Spatial variability of brightness temperature (*T_b_*) (**a**,**d**), corrected *T_b_* (**b**,**e**) and LST (*T_s_*) (**c**,**f**) in the FLIR camera footprint area with embedded thermocouples on 9 December, 16.50 hours and 8 December, 12.05 h, 2015.

To demonstrate the spatial variation in surface temperature over the target area containing the embedded TC’s on the surface, *T_b_* within the footprint area of FLIR is shown in Figure 4a,e. The average *T_b_* on 9 December at 16.50 LST and 8 December 2015 at 12.15 LST were 26.48 (mean) ± 0.58 (S.D) and 16.43 ± 0.21 °C, respectively. The homogeneity of FLIR footprint was affected by the presence of TC cables, even though the cables were coated with the same material and were embedded in the surface as shown in Figure 4. To evaluate the effect of the TC cables on *T_b_*, an average of *T_b_* values within 100 × 100 pixels for an area devoid of cables, between the TC cable locations was carried out and they were 26.48 ± 0.33 and 16.40 ± 0.05 °C, respectively, suggesting a reduction in variability of *T_b_* over that area. Overall, the magnitudes of BTs by FLIR were higher than those by IRTs as shown in Figure 2 and Table 3. The linear regression analysis between *T_b_* by IRTs and FLIR indicated a close agreement with a slope of 1.03 and an intercept of >−3.9 °C. To correct this systematic bias, the offset of 3.9 obtained from the average of the three IRTs were reduced from *T_b_* for each pixel and the effect of this correction on the spatial variability of BTs are shown in Figure 4b,e. These corrected values of BTs were used to estimate LST (Figure 4c,f using Equation (2)). After this correction, the relationship between mean BTs by IRTs and FLIR measurements were (*y* = 1.03*x* + 0.08, *r*^2^ = 0.99, Bias = 0.522, STDd = 0.5, RMSE = 0.71 °C) similar to the IRTs. This demonstrates the use of IRTs to assess any systematic bias of FLIR based *T_b_* measurements. Even though the FOV of FLIR appeared homogeneous, the difference between maximum and minimum values of LSTs within the footprint, after excluding the pixels containing the embedded TC cables, varied from 0.5 to 4 °C, while standard deviation from the mean varied from 0.02 to 0.75 °C with highest values during noon time clear-sky conditions.

### 3.2. Comparison of LST Measurements Using All In Situ Sensors

**Figure 5:**
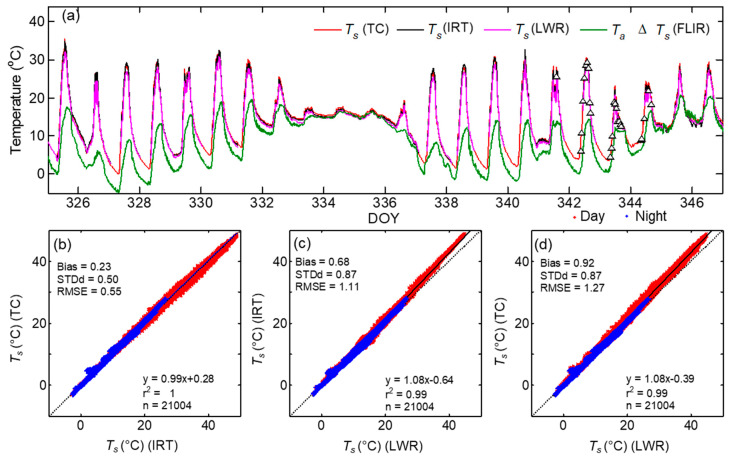
(**a**) Time series of 5-min LST (*T_s_*) by thermocouples (TC), IRTs, longwave radiation measurements (LWR), FLIR camera, and air temperature during DOY 325–347. The linear regression (solid line) between LST measurements, (**b**) *T_s_* (TC) vs. *T_s_* (IRT), (**c**) *T_s_* (IRT) vs. *T_s_* (LWR), and (**d**) *T_s_* (TC) vs. *T_s_* (LWR) for the whole dataset are shown.

To evaluate how the LSTs measured by IRT’s, TC, and FLIR compare with those estimated from longwave radiation (LWR) measurements by CNR, the time series of LSTs are shown in Figure 5a and corresponding linear regression analysis is shown in Figure 5b–d. Here the mean values of the LST by the three IRTs were used. As mentioned above the surface temperature measured by IRT sensors and imagers are brightness temperatures rather than actual *T_s_*. So, it must be corrected for surface emissivity and reflectivity effects to get the true estimates of *T_s_*. During the experiment we noticed the effect of dew deposition on incoming longwave radiation measurements which appeared as a spike [54], especially during early morning hours for a few days and these data points were removed leading to gaps in LSTs estimated using Equations (2) and (3). After the emissivity and reflectivity correction, the LSTs by IRTs, FLIR, and LWR agreed very well in magnitude with the direct measurements of *T_s_* by TC with *r*^2^ > 0.99 (Figure 5 and Table 4). The difference between *T_s_* (LWR) and other LST measurements were noticeable mainly during daytime. The mean bias (0.23 °C), STDd (0.50 °C), and RMSE (0.55 °C) from the comparison of *T_s_* (TC) with mean *T_s_* by IRTs was smaller than the bias and RMSE obtained using *T_s_* (IRT) or *T_s_* (TC) with *T_s_* (LWR) (bias = 0.68, STDd = 0.87 RMSE = 1.11 °C; and bias = 0.92, STDd = 0.87 and RMSE = 1.27 °C), respectively. This clearly indicates that the higher biases between LST measurements were contributed by *T_s_* (LWR). The bias, STDd, and RMSE between the LST measurements by the three methods were higher during daytime than those during nighttime (Table 4) because the Earth’s surface is more thermally homogeneous during nighttime [55]. Due to this, the absolute bias between nighttime *T_s_* measurements by the three methods above resulted in a value <0.5 °C, anticipated accuracy for ground-based LST measurements.

The mean LSTs by IRTs and the corrected daytime LSTs by FLIR also agreed well (*y* = 0.98*x* + 0.37, *r*^2^ = 0.99, bias = 0.0.03, STDd = 0.65, RMSE = 0.64 °C) However, during daytime clear sky conditions the *T_s_* (IRT) and *T_s_* (TC) was higher than *T_s_* (LWR) by 1.7 and 1.9 °C, respectively. Similar to BTs, the in situ LSTs by IRTs agreed well with each other. The mean bias between *T_s_* by JPLR and Apogee were (bias = 0.0001 with STDd = 0.16 and RMSE = 0.16 °C) smaller than those between Heitronics and Apogee sensors (bias = −0.28 with STDd = 0.27, RMSE = 0.39 °C) or JPLR and Heitronics (bias = 0.26 with STDd = 0.29, RMSE = 0.40 °C). As the footprint of the downward looking pyrgeometer is a larger area than the surface prepared for this experiment, it is most likely that the homogeneity of the footprint was affected by part of the tripod base and the regular asphalt pavement in the parking lot, outside the freshly prepared area. The differential heating due to the emissivity difference might have contributed to higher bias of daytime *T_s_* (LWR).

### 3.3. Comparison of Land Surface Temperature and Near Surface Air Temperature

**Figure 6:**
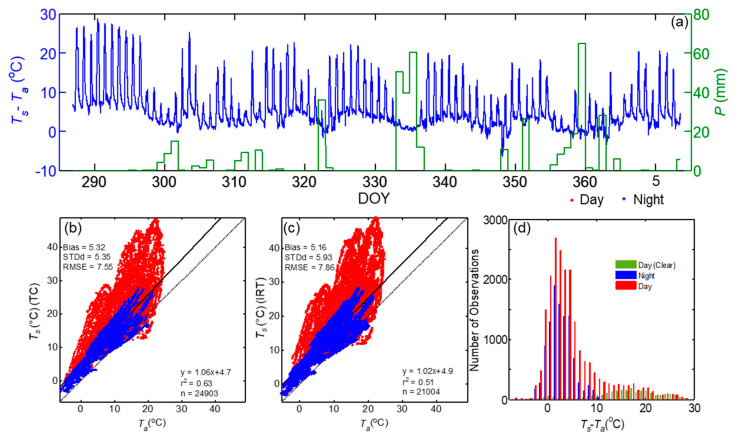
(**a**) Times series of the difference between mean LSTs by IRTs (*T_s_*) and mean air temperature (AT) by platinum resistance thermometers (PRTs) and daily total precipitation (*P*) during the campaign period, comparison of 5-min mean LST measurement by (**b**) thermocouples and (**c**) IRTs vs. air temperature (*T_a_*). Histogram of the difference between LST (IRT) and AT for different periods are also shown (**d**).

The time series of near surface air temperature (*T_a_*) during DOY 325–347 and the difference between mean LST by IRTs and AT for the study period are shown in Figure 5 and Figure 6, respectively, to examine how the LSTs by multiple sensors compare with AT. During the entire period, LST was consistently higher than *T_a_* with a few exceptions during cloudy or rainy periods. The temporal variation of mean LST and AT, indicate that AT was lower than LST by 30 °C during precipitation free clear days than those during night time or rainy days. 

During the observation period, mean LST by IRTs varied from −2.9 to 48.9 °C, and *T_a_* varied from −6.3 to 24.7 °C. The difference between *T_s_* (IRT) and *T_a_* varied from −7.3 to 29 °C. To better evaluate the magnitudes of AT and LST, a regression analysis was performed (Figure 6b,c and Table 5). *T_a_* explained 63% (51%) of the variance in *T_s_* (TC) (*T_s_* (IRT)) and had a bias of 5.32 (5.16), STDd of 5.35 (5.93) and RMSE of 7.55 °C (7.86 °C). The LST showed a drastic increase when *T_a_* reached 20 °C or above and resulted in a non-linear relationship above that limit whereas the relationship between *T_s_* and *T_a_* was linear and statistically better during nighttime conditions. The distribution of the differences between *T_s_* and *T_a_* are shown in Figure 6d, indicating that during daytime clear sky conditions *T_s_* was well above *T_a_* by 10 to 30 °C. The difference between *T_s_* (TC) and *T_a_* during nighttime was 3.22 °C with *r*^2^ > above 0.87.

### 3.4. Comparison of LST Measurements over Four Grassland Sites

**Figure 7:**
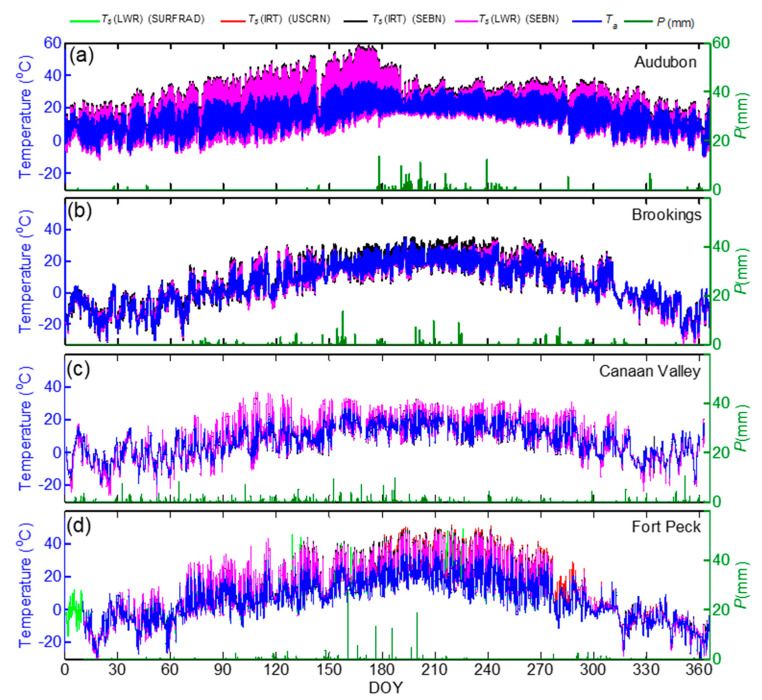
Time series of 30-min air temperature (*T_a_*), precipitation (*P*), and land surface temperature (*T_s_*) by IRT and LWR measurements at the SEBN sites. LST measurements from the nearby United States (**a**–**d**) Climate Reference Network (USCRN) and (Surface Radiation Network) SURFRAD sites are included on panel 4 for the Fort Peck site.

As both the direct measurement of LST by thermocouple and directional measurements by IRT showed noticeable difference from LST by hemispherical longwave radiation measurements over the parking lot, especially during daytime, we extended the comparison of *T_s_* (LWR), *T_s_* (IRT), and *T_a_* measurements to four grassland sites (Figure 7). 

**Figure 8:**
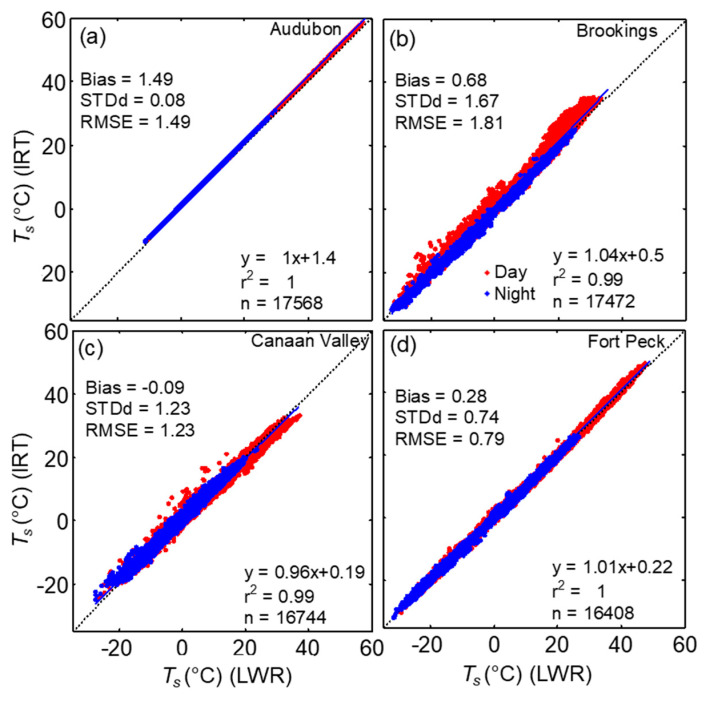
Comparison half-hourly LST measured by IRTs (*T_s_* (IRT)) with LST estimated by using longwave radiation measurements (*T_s_* (LWR)) at (**a**) Audubon, (**b**) Brookings, (**c**) Canaan Valley, and (**d**) Fort Peck grasslands sites. The linear regression (solid line) for the whole dataset and the results are shown. The dotted line indicates 1:1 relationship between the variables (See Table 6).

As the incoming longwave measurement at the Fort Peck SEBN site had issues during the first half of the year, similar measurements from the collocated SURFRAD site were used to estimate LST at Fort Peck site. The comparison of upwelling longwave radiation measurements at both SEBN and SURFRAD sites showed very good agreement; LWR_up_ (SEBN) = 1.04(LWR_up_ (SURFRAD))-14, *r*^2^ = 1.99, *n* = 17116, with a bias of 2.05, RMSE = 7.78, and STDd = 7.5 Wm^−2^. The bias and RMSE was higher during daytime periods (3.6, 10.44 Wm^−2^, respectively) than those during nighttime periods (0.50 and 3.66 Wm^−2^, respectively). The annual cycles of surface temperature at the four sites indicate weather with cool winters and warm summers with the exception mainly due to the precipitation distribution at each site (Figure 7). The highest LST was recorded at the Audubon site (59 °C) followed by Fort Peck (49 °C). The time series of *T_s_* (IRT) and *T_s_* (LWR) showed close agreement at the four sites and the results of linear regression analysis for the entire dataset are shown in Figure 8 and Table 6. The absolute difference between *T_s_* (IRT) and *T_s_* (LWR) for the annual data was <0.7 °C, except at the Audubon site (1.49 °C) and the correlations coefficient was above 0.99. At the Fort Peck site, the relationship between *T_s_* (LWR) at SEBN and SURFRAD sites was better than the relationship between *T_s_* (IRT) at SEBN and *T_s_* (LWR) at SURFRAD. During DOY 175–366, *T_s_* (IRT) at USCRN site was higher than *T_s_* (LWR) at SURFRAD, *T_s_* (IRT) or *T_s_* (LWR) at SEBN site. The slope of the regression, bias, STDd, and RMSE performed better, with a few exceptions, during nighttime conditions at all sites, due to better thermal homogeneity (Table 6).

**Figure 9:**
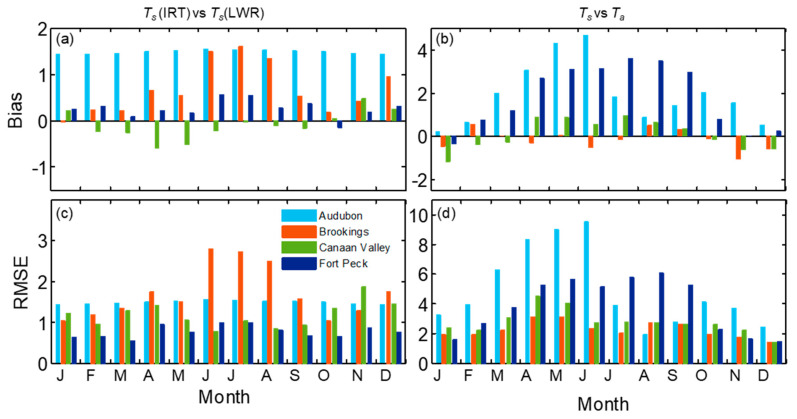
Monthly values of bias and root mean square error (RMSE) for *T_s_* (IRT) vs. *T_s_* (LWR) (**a**,**c**); and *T_s_* (IRT) *T_a_* (**b**,**d**), respectively for the four grassland sites.

To explore further on how the relationship between LST measurements vary seasonally, the results of monthly statistics are shown in Figure 9a,c. The magnitudes of *T_s_* (IRT) were higher than *T_s_* (LWR) for all sites except Canaan Valley. The monthly bias showed a transition during the beginning and end of the growing season in spring (April–May) and November, respectively, at this site. The bias and RMSE obtained from the comparison of *T_s_* (IRT) and *T_s_* (LWR), indicated strong seasonal variation at Brookings and Fort Peck. In these sites, bias was higher during the peak growing season from June to August when the vegetation growth reached its highest of the season [56,57]. The bias and RMSE at the Brookings were 1.6 and 2.7 °C in August, whereas at Fort Peck it was >0.55 °C and ~1 °C in June and July, respectively. However, at Canaan Valley, the absolute difference between *T_s_* (IRT) and *T_s_* (LWR) was lower than 0.58 °C throughout the year with lower values of RMSE during May to September. At Audubon semiarid grassland, the bias and RMSE were consistently high throughout the year (both > 1.4 °C), with slightly higher values (>1.52 °C) during May–September. However, monthly values of STDd (not shown here) from the comparison of *T_s_* (IRT) and *T_s_* (LWR) were lower than <0.09 °C during the year at the Audubon site, whereas it varied from 0.5 to 2.3 °C for the other sites. However, the distribution of the difference between half-hourly daytime *T_s_* (IRT) and *T_s_* (LWR) in Figure 9 indicates a large difference, exceeding 5 °C during daytime in Brookings followed by Canaan Valley whereas in the other three sites it was mostly less than 2 °C.

**Figure 10:**
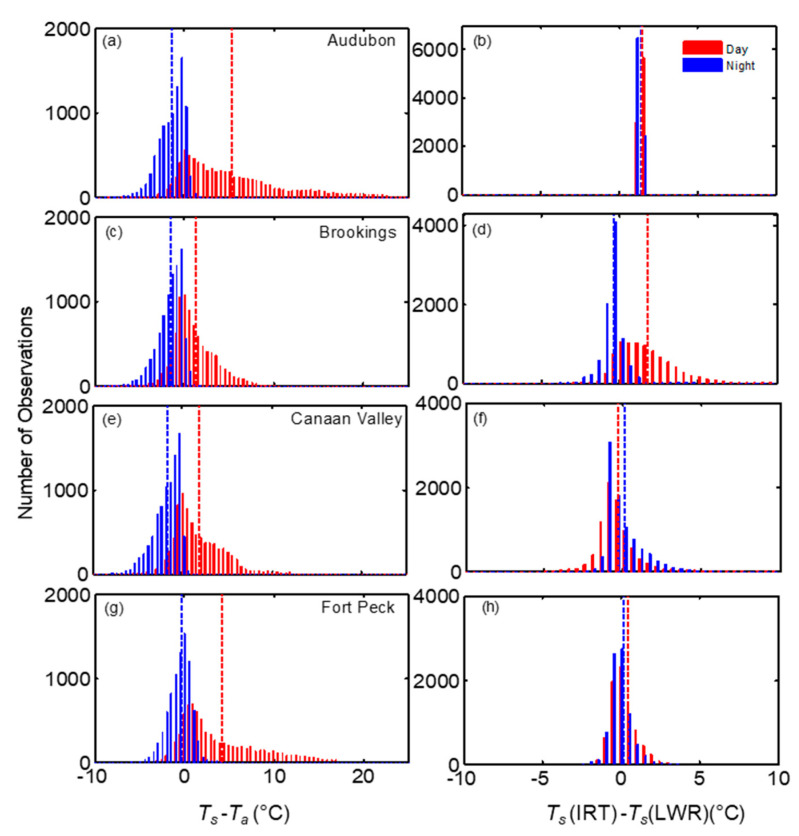
Histograms of the difference between *T_s_* (IRT) and *T_a_* (**a**,**c**,**e**,**g**), and *T_s_* (IRT)-*T_s_*(LWR) (**b**,**d**,**f**,**h**) during daytime and nighttime periods for the four grassland sites. Corresponding mean values are shown by blue and red dotted lines.

**Figure 11:**
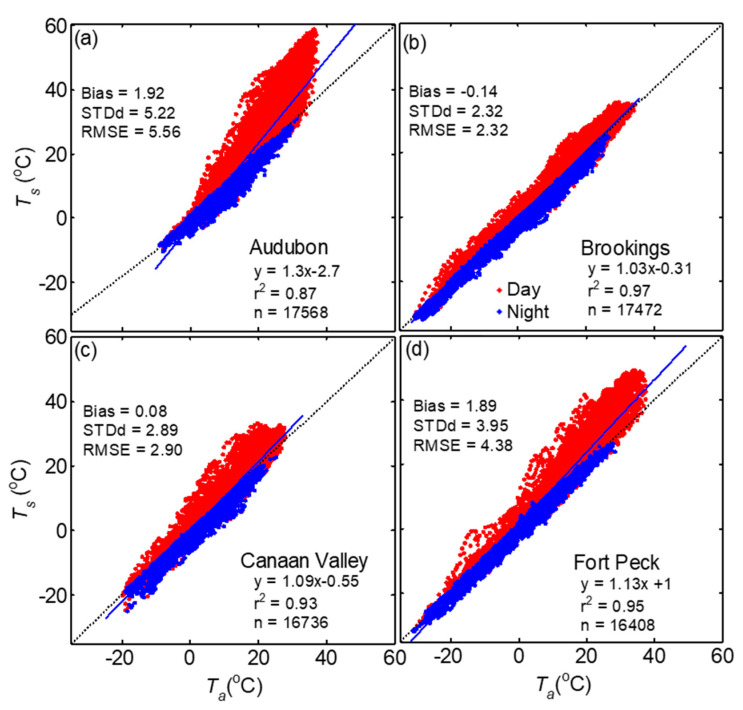
Comparison of LST measured by IRTs (*T_s_*) with AT measurements (*T_a_*) at (**a**) Audubon, (**b**) Brookings, (**c**) Canaan Valley, and (**d**) Fort Peck grasslands sites. The results of the linear regression (solid line) for the whole dataset are shown (see Table 6).

Among the four sites, the magnitudes of daytime half hourly *T_s_*-*T_a_* at the Audubon site reached up to 25 °C followed by Fort Peck and Canaan Valley (<20 °C) (Figure 7 and Figure 10), whereas during nighttime *T_s_* was mostly lower than *T_a_* by <5 °C. The difference between *T_s_* and *T_a_* for annual dataset was highest at Fort Peck (1.9 °C) and Audubon (1.9 °C) with RMSE 4.38 and 5.56 °C, respectively (Figure 11 and Table 6). At Canaan Valley and Brookings, the bias was 0.08 and 0.14 °C, respectively. The RMSE between *T_s_* and *T_a_* were among the highest at Audubon and Fort Peck during daytime conditions, but it was the highest at Brookings and Canaan Valley during nighttime periods. However, on a monthly basis, all sites showed distinct patterns (Figure 9b,d), but mostly with larger *T_s_*-*T_a_* values during peak summer months. At the Audubon site *T_s_*-*T_a_* and RMSE reached its peak values in June (4.7 and 9 °C, respectively), and these values reduced drastically by July following the onset of North American monsoon season (Figure 7a) and increase in vegetative activity [58]. The bias and RMSE between *T_s_* and *T_a_* at Fort Peck were consistently above 2.9 and 5.0 °C, respectively, during May to September. Whereas at Brookings, both *T_s_* and *T_a_* agreed well during most of the year (Figure 7b), especially during June to September compared with other sites with monthly absolute bias < 0.5 and RMSE < 2.7 °C, but higher values during spring and fall months. The bias was <1 °C at Canaan valley but RMSE reached values close to 4 °C during June to July. As expected, *T_a_* was mostly higher than *T_s_* during winter months.

## 4. Discussion and Conclusions

Our results show that the ground-based surface BTs by the three IRTs over the entire period agreed quite well within <0.3 °C with STDd < 0.27 °C and RMSE < 0.36 °C confirming that these instruments are suitable for short and long-term studies on land surface interaction as well as for providing high quality validation data for satellite and other applications [19]. As LSTs are indirect measurements, accuracy of LST can be influenced by the accuracy of *ε* and downwelling irradiance. However, its effect on the intercomparison experiment here is minimal due to the same values of *ε* and *L*_↓_ used for the estimation of LSTs by all sensors. After the correction, the estimated LSTs by IRTs were in very good agreement with LSTs by TCs with an absolute difference of 0.23 °C with STDd of 0.50 and RMSE of 0.55 °C. The in situ LSTs by the IRTs also agree well with each other with an absolute bias of <0.3 and RMSE < 0.4 °C. These values are slightly lower than the average absolute deviation of 0.44 °C from the mean and an average standard deviation of 0.18 °C between the in situ LSTs by five IR radiometers on the gravel plains near Gobabeb Training and Research Centre in Namibia [33]. The direct measurement of LST by TCs, at actual surface emissivity of 0.9 were higher than the BTs by IRTs for ε = 1 by ~2 °C over the parking lot surface, demonstrate the impact of radiance and emissivity correction (Section 2) on the magnitude of LST. Because of the higher values of ε close to 1, this effect was small at the grassland sites. At the grassland sites, the absolute difference between LST and BT for the annual data were <0.15 °C for sites with ε of 0.987, whereas it was ~0.57 °C for Audubon grassland (ε = 0.975). These results agree with our previous study over a grassland USCRN site showed that LST is less sensitive to *L*_↓_ than ε and for a range values of ε between 0.9 and 1, an increase in ε by 1% (within the range 0.95–1) resulted in an average decrease of LST by 0.17 ± 0.04 °C for similar values of *L*_↓_ [44]. Based on measurements over a rice field, [59] reported that an uncertainty of 0.2–0.4 °C can be expected for an uncertainty of 0.01 in emissivity.

Among the three types of in situ sensors, including TCs, IRTs, and pyrgeometer, there was better agreement between the LSTs by three different IRTs (<0.3 °C) than between the LSTs by pyrgeometers and any IRT (>0.67 °C). The comparison of LST (TC) with LST (IRT) also yielded better results than the comparison of LST (IRT) and LST (LWR) during the entire study period including daytime clear-sky conditions. The difference between LST (IRT) and LST (LWR) was ~2 °C during clear-sky conditions over the parking lot. Over the grassland sites, the monthly difference between LST (IRT) and LST (LWR) reached up to 2 °C, depending up on the heterogeneity of the site and the season [60,61]. As TCs provides point measurements of LST, and multiple TCs can provide an average value of LST of the target. Here, we have used TC as a direct method to measure LST by embedding it to the asphalt surface. However, continuous measurements using TCs in field studies is very challenging, as it can detach after installation and also malfunction (Section 2). Additional uncertainties in temperature measurements using TCs can result from other factors like cable drift, spurious junction voltages, inadequate voltmeter sensitivity, and reference temperature uncertainty [62]. Although, contact sensors like TCs can provide leaf or tree temperature during short-term intensive experiment [63,64], LST measurements over vegetative canopies are not reliable as the point measurement by TC is less representative of the field site compared with non-contact thermal infrared sensors over an area. Over dense vegetation like forest areas, the tower, airborne, or satellite-based LST measurement by IR sensors usually provide the top canopy temperature rather than soil surface or understory temperature.

By definition, LST is a thermodynamic temperature that can be felt or measured by an accurate thermometer at the land surface-atmosphere point-of-contact and is independent of wavelength [9,53]. This can be equivalent to the ensemble directional radiometric temperature only for isothermal and homogeneous surfaces [8,9]. Practically, LST is derived by in situ or remote sensing instruments, using the thermal radiance coming from the surface in a finite wavelength band within the FOV in the direction of the sensor. The uncertainties in the comparison of LST measurements by various IR sensors can occur due to the difference in the accuracy and precision of the in situ sensors, differences in measurement techniques for target BTs, differences in FOV, wavelength bands, and spectral response functions of the sensors. The accuracy of the LSTs by IRT sensors given by the manufacturers are <±0.5 °C, which agrees with the results presented here, whereas for longwave radiation measurements it is up to ±10% of daily totals. However, field studies on intercomparison of longwave radiations measurements using multiple radiometers including CNR1 net radiometer during the energy Balance Experiment (EBEX-2000) showed that the accuracy of incoming and outgoing longwave radiations vary up to 10 Wm^−2^ during daytime and 5 Wm^−2^ during night time. Similar comparison during HiWATER experiment using Eppley Precision IR radiometer as reference revealed larger difference in longwave radiation during daytime especially around noon time (8 Wm^−2^) and 3 Wm^−2^ during nighttime equivalent to an error of 1.2K in the LST at daytime and 0.5 K in the LST at nighttime, respectively [65,66]. Similar comparison of outgoing longwave radiation at Fort Peck revealed higher bias during daytime (3.6 Wm^−2^) than nighttime periods (0.50 Wm^−2^) demonstrating the better agreement of LSTs during nighttime periods.

The footprint of the ground-based sensors used in this study can vary up to 1 to 2 orders due to the sensor’s FOV, angle, and height of the measurement. The effects of FOV on measured LST’s can be more pronounced in the comparison of directional IRTs and hemispherical pyrgeometers. If the sensors are mounted anywhere between 1 to 10 m height, the footprint of narrow angle radiometers like Apogee and JPLR with half-angle FOV ~20° can result in circles with a diameter ranging from ~1 to 7.5 m while the hemispherical measurements by pyrgeometers with FOV ~150° can cover a circular area with a diameter of ~7 to 75 m. To view similar area of the target like the other IRTs, the narrow angle IR radiometers like Heitronics should be mounted above the ground by ~15 to ~150 m making it more suitable to be used from an aerial platform. There was better agreement between JPLR and Apogee BTs (< ±0.003 °C) than those with Heitronics BTs (<0.26 °C) because of the similar wavelength range, FOV, and footprint area. The experimental area was spatially homogeneous and was large enough that it exceeded the target area viewed by the IRTs. However, the measurements by thermal imager showed that there were apparent variations in LST over the surface most likely due to the changes in emissivity distribution due to the small-scale difference in the surface characteristics [49]. As the measurement by different IRTs depends on the spatial frequency of the temperature variations on the area of the target in their FOV, ideally all participating radiometers should observe the same area of the target [32]. As the Heitronics sensor footprints (<0.1 m in diameter at 1.7 m instrument height) were one order smaller than those by JPLR and Apogee (>1 m in diameter), the areas monitored by these radiometers will be of comparable size if the Heitronics sensor was mounted at ~25 m at nadir above the ground. As these can lead to the presence of installation components in the FOV of this sensor and the pyrgeometer, all the sensors were mounted at the same height adjacent to each other with the footprint overlapping each other, so that they can cover different areas of the same target, homogeneous and isothermal as possible, in their FOV.

Usually the IRTs or pyrgeometers are mounted vertically, pointing downward on a long boom that is usually a few meters in length. This helps to prevent contamination of the footprint of the sensor by the tower installation parts, especially IRTs if mounted at lower heights (<2 m), like in the USCRN network and the grassland sites used here. However, this issue cannot be avoided if the directional narrow bands IRTs are many meters above the canopy or in the case of hemispherical pyrgeometers if the mounting height is above a few meters, which is typical for of many FLUXNET sites that use four-way radiometers. One way to prevent this is to install IR radiometers at a near-nadir view angle (<30°) in field experiments [32,33], but not in the case of four-way radiometers. Based on simulations, [67] found that for a sensor with a narrow FOV in the nadir of the urban surface, directional radiometric temperature differs from actual LST by <±1.9 K, whereas it was <±2.9 K for off-nadir view directions with highest values during daytime. Over a semiarid grassland, [68] reported that the difference between nadir and off nadir radiative temperature varied up to 5 K, especially when biomass reached its maximum suggesting the directional effects on LSTs. In this study, LSTs using pyrgeometers measurements were lower than those using IRTs with a few exceptions, as a wider angle might lead to lower surface temperatures for the same land cover even though the effect is small [69]. The effects of FOV on LST measurements will be small if the target is homogeneous with negligible anisotropy [53]. However, FLIR images over a visually homogeneous asphalt surfaces suggest standard deviations of LST for the study period <0.75 °C, but the difference between maximum and minimum values of LSTs varied from 0.5 to 4 °C. This agrees with the report by [32] on the apparent surface temperature variations on homogeneous looking samples of shortgrass (up to 5 °C), clover (10 °C), gravel (10 °C), dark soil (10 °C), sand (5 °C), and asphalt (3 °C) based on thermal images. The temperature variations of the surface can occur due to the spatial variations in surface characteristics including surface roughness, emissivity, thermal conductivity, reflectivity, structure, and small-scale topography. Over the grassland sites the spatial variations in LST within the footprint of the sensors are inevitable due to the presence of soil, changes in vegetation, soil moisture, and its seasonal evolution resulting in heterogeneities and anisotropy within the FOV of sensors. Our study revealed a larger difference between seasonal and annual values of LST (IRT) and LST (LWR) at Audubon (<2 °C), a semiarid grassland with growing season vegetation cover was ~40% in [58]. Similar differences (~2 °C) in LST (IRT) and LST (LWR) were observed by [60] over a vineyard with exposed soil than over a homogeneous grassland site (0.3 °C) during daytime conditions. During the warmer growing season, there can be maximum contrast between the dry soil and active transpiring vegetation leading to larger difference in monthly LST estimates in most of the grasslands and it was more pronounced in Audubon, Brookings, and Fort Peck, even though its magnitude can be affected by the seasonal distribution of precipitation and soil water content. For the Fort Peck and Canaan valley grasslands, both RMSE and bias for LST (IRT) and LST (LWR) were mostly <±1 °C. However, the distribution of half hourly LST (IRT)-LST (LWR), showed that the difference in these measurements can exceed 5 °C, especially at Brookings and Canaan Valley sites, suggesting heterogeneities and anisotropies in the FOV of both sensors. With the increase in duration or spatial coverage of the data the positive and negative biases resulting from short-term changes in environmental conditions or small-scale heterogeneous within FOV, possibly could offset leading to smaller mean bias in the comparison of LSTs. Ideally, the uncertainties in ground-based LST measurements should be <±1 °C for validation of satellite data and for assessing the performance of numerical models [70]. For the above uses, it is preferable to carry out LST measurements over homogeneous surfaces and by ensuring large footprints, which can made possible by using pyrgeometers because of its stable performance and larger footprints or by raising the IRT to higher levels without contaminating the footprint with tower installation parts. Our results show that large uncertainties (>1 to 2 °C) in situ LST measurements of the same order reported for satellite-based LST measurements can occur in daytime conditions resulting from the surface heterogeneities depending upon the site, its characteristics and changes in vegetation phenology, if any. However, during nighttime, LST measurements by all sensors agreed better over all sites due to thermally homogeneous conditions during night. This suggests that the validation results or comparison of LST between different platforms can vary based on the measurement methodology used for in situ measurements, difference in accuracy of the sensor, FOV, surface characteristics of the target, time of the day, sky conditions, and seasonal vegetation characteristics, if any, depending on the site.

Over the asphalt surface in the parking lot, LST was higher than AT during the observational period (>12 °C during daytime and <2 °C during nighttime) with mid-day values exceeding 25 °C during clear air conditions agreeing with the results of [71] over various urban land covers including asphalt surface. The values of *T_s_*-*T_a_* were generally larger for the non-precipitating days than for the precipitating days. At grassland sites nighttime *T_a_* was mostly greater than LST. This was expected, and is consistent with the reports over vegetated surfaces [51,72,73]. Whereas during the daytime, the LST was higher than *T_a_* and it varied from 0.86 to ~4.98 °C with highest values at Audubon for the entire dataset. This difference is within the range of values reported by [27] and [74] over many USCRN sites. Nighttime AT was a more reliable proxy for LST than daytime LST [12]. The comparison of annual and monthly LST and AT over grassland sites indicate that the difference in these two temperatures depend on the site, time of the day, sky conditions, soil moisture, vegetation growth, and the season [11]. This result agrees with the earlier studies on the comparison of satellite LST with AT from spatially and temporally collocated sites [24,25,26,44]. Understanding the relationship between LST and AT over different ecosystems is also required for deriving satellite-based AT from LST [12,13,14]. Both LST and AT, from in situ and remote sensing platforms are needed to evaluate the accuracy of the simulation of near-surface atmospheric diurnal variation, one of the difficult and most important task of numerical weather prediction and in the improvement of model performance [11,16,17,75]. The utilization of in situ LST data for satellite LST validation is already demonstrated by many researchers [7,23,41,44,53,57,59,70]; however, a previous study reported that irrespective of the use of daytime or nighttime data, the use of AT instead of LST in the comparison of ground and satellite LST can result in an increased bias and RMSE [44].

The thermal images by FLIR camera, even though only covering a few days during the campaign period, clearly shows the capability of the IR imager to capture the spatial and diurnal variation of LST in very good agreement with other in situ sensors. This information captured over a large area using the imagers onboard UAV or aircraft, is very useful in the study of land-atmospheric interaction, hydrology, and agriculture at spatial scales larger than ground measurements but at scales that are unable to be replicated by satellite platforms [76]. However, most of the IR imagers have low manufacturers stated accuracy (>±2.0 °C) compared to IRTs (<±0.5 °C) and are suited mainly for short-term experiments or for aerial flight campaigns. The calibrated uncooled microbolometer thermal infrared cameras, like the one used here, have been reported to perform well in stable laboratory conditions with accuracy <±0.5 °C, but under changing ambient field conditions the accuracy can decline to >±5.0 °C [36,77]. The linear regression analysis between BTs measured by IRTs and FLIR over the parking lot revealed an offset of ~3.9 °C. This systematic bias did not change even after removing the pixels that contain the embedded cables for TCs. It was within the order of the difference in temperature reported using thermal imager and IR sensors in the field studies, for example 6.06 °C by [38] over a glacier and between 1.5 and 5 °C by [76]; over various crops surface under different stages of cultivation. Surface heterogeneity or difference in FOV can contribute partially to the bias in temperatures, but correcting the significant systematic bias for each pixel is important in many applications that use airborne measurements of LST. For example, even a few degrees bias in LST can lead to significant error in the estimated energy fluxes using high resolution of the thermal images using aerial platforms like UAVs [78,79,80]. One way is to use high accuracy low cost IRTs along with IR cameras onboard to calibrate the BT measurements [81] as demonstrated here. After the offset correction, the estimated LST’s agree very well with the LSTs by other IR sensors. Due to its narrow FOV, the Heitronics or similar sensors are often used in airborne measurements [44,75], and similar IRTs can be used to calibrate the BTs by IR imagers in use. Additional errors in LSTs by thermal images onboard UAV or manned aircraft can occur if the atmospheric correction is not taken to account especially above 150 m [82,83,84], but in this study its effect is negligible due to low mounting height of the imager. There are additional sources of errors in deriving LST from TIR cameras such as vignetting, non-uniformity noise, radiometric calibration, and sensor temperature. Of these, the poor performance mostly results from the non-linear relationship between camera output and sensor temperature and it can be up to ±20 °C [36,77]. Further work is needed to improve the overall accuracy, resolution, and performance of thermal IR cameras for applications that need accuracy and precision of LST similar to IRT sensors.

## Figures and Tables

**Table 1:** Specifications of the thermal infrared (IR) radiometers.

Instrument	Spectral Range (µm)	Accuracy	Instrument Height (m)	FOV (°)	Footprint Area (m^2^)
**Infrared Thermometer (IRT)**		(°C)			
Apogee (SI-111)	8–14	±0.2 ^3^	1.7	44	1.48
Apogee (IRTS-P)	6.5–14	±0.3	2.0	56	3.55
Heitronics (KT19.85)	9.6–1.5	±0.2	1.7	3	0.006
JPLR (500 series) ^1^	8–14	±0.1	1.7	36	0.96
**IR camera**					
FLIR (Tau2)	7.5–13.0	±5.0	1.7	45 × 35	1.51
**Pyrgeometer (LWR) ^2^**		(Wm^−2^)			
Kipp and Zonen (CNR1-CG3)	4.5–42.0	DT ± 10% ^4^	1.7	150	126

^1^ For JPL Quasi nulling radiometer (JPLR), the accuracy is for the calibrated range from 4 to 40 °C. ^2^ The acronym LWR is used to indicate land surface temperature (LST) estimation by using longwave radiation measurements. ^3^ Accuracy is ±0.2 °C for the temperature range −10 °C to +65 °C; and ±0.5 °C for −40 °C to +70 °C. ^4^ DT indicates daily totals.

**Table 2:** Characteristics of the field sites.

Site	Latitude, Longitude	Elevation (m)	Year	Measurement Height (m)	
				LST	AT
Audubon, Arizona	31.5907 N, 110.5090 W	1469	2008	2	1.25
Brookings, South Dakota	44.3452 N, 96.8358 W	497	2008	2	1.5
Canaan Valley, West Virginia	39.0633 N, 79.4208 W	994	2008	2	2.5
Fort Peck, Montana	48.3077 N, 105.1019 W	634	2012	2	1.25

**Table 3:** Comparison of surface temperature measurements (*T_b_* or *T_s_*) by in situ sensors during 10 October 2015 to 8 January 2016. Bias, standard deviation of the difference (STDd), root mean square error (RMSE) in °C, and results of the linear regression analysis (*y* = *bx* + *c*) ^1^ are presented.

Data	*y*	*x*	(*b*, *c*)	*r* ^2^	RMSE	Bias	STDd	*n*
All	*T_b_* (Heitronics)	*T_b_* (Apogee)	1.01, −0.37	1	0.36	−0.26	0.25	24,914
Daytime			0.99, −0.2	1	0.34	−0.22	0.25	11,447
Nighttime			1.02, −0.52	1	0.38	−0.29	0.24	13,468
All	*T_b_* (JPLR)	*T_b_* (Heitronics)	0.99, 0.37	1	0.35	0.23	0.27	21,860
Daytime			1, 0.11	1	0.29	0.15	0.25	10,801
Nighttime			0.98, 0.6	1	0.41	0.30	0.27	11,059
All	*T_b_* (JPLR)	*T_b_* (Apogee)	0.99, 0.8	1	0.16	−0.003	0.16	21,860
Daytime			0.99, −0.03	1	0.21	−0.06	0.19	10,801
Nighttime			0.99, 0.08	1	0.09	0.05	0.08	11,059
All	*T_b_* (Heitronics)	*T_s_* (TC)	0.97, −1.5	0.98	2.25	−1.95	1.14	24,914
Daytime			0.93, −0.65	0.98	2.53	−2.14	1.34	11,447
Nighttime			01.07, 2.6	0.98	1.99	−1.78	0.89	13,468
All	*T_b_* (JPLR)	*T_s_* (TC)	0.93, −0.47	0.98	1.92	−1.63	0.97	21,860
Daytime			0.91, −0.12	0.98	2.28	−1.94	1.21	10,801
Nighttime			1.04, −1.9	0.98	1.48	−1.32	0.65	11,059
All	*T_b_* (Apogee)	*T_s_* (TC)	0.96, −1.1	0.99	1.94	−1.68	0.97	24,914
Daytime			0.93, −0.49	0.99	2.25	−1.91	1.19	11,447
Nighttime			1.05, −2	0.99	1.63	−1.48	0.67	13,468
All	Mean *T_b_* (IRTs)	*T_b_* (FLIR)	1.03, −3.9	0.99	3.41	−3.37	0.49	25

^1^ Here *y* is the independent variable, and *x* is the dependent variable, *b* and *c* are the slope, and intercept of the linear equation. Bias, STDd, and RMSE are estimated using ∆*T* = *y* − *x*. The coefficient of correlation (*r*^2^) and the numbers of observations (*n*) are also included.

**Table 4:** Comparison of land surface temperature (*T_s_*) by different sensors. Bias, STDd, RMSE in °C, and results of the linear regression analysis (*y* = *bx* + *c*) ^1^ are presented.

Data	*y*	*x*	(*b*, *c*)	*r* ^2^	RMSE	Bias	STDd	*n*
All	*T_s_*(TC)	*T_s_*(IRT)	0.99, 0.28	1	0.55	0.23	0.50	21,004
Daytime			0.98, 0.59	1	0.67	0.26	0.62	10,509
Nighttime			1.02, −0.05	1	0.40	0.40	0.20	10,495
Day clear sky			0.97, 0.86	1	0.81	0.23	0.78	3532
All	*T_s_*(TC)	*T_s_*(LWR)	1.08, 0.−39	1	1.27	0.92	0.88	21,004
Daytime			1.07, −0.15	0.99	1.65	1.36	0.94	10,509
Nighttime			1.05, −0.12	0.99	0.70	0.47	0.52	10,495
Day clear sky			1.08, 0.11	0.99	2.21	1.91	1.10	3532
All	*T_s_*(IRT)	*T_s_*(LWR)	1.08, −0.64	0.99	1.11	0.68	0.87	21,004
Daytime			1.09, −0.72	0.99	1.46	1.09	0.96	10,509
Nighttime			1.02, −0.034	0.99	0.56	0.27	0.49	10,495
Day clear sky			1.1, −0.75	1	2.05	1.68	1.16	3532

^1^ Same as in Table 3.

**Table 5:** Comparison of land surface temperature (*T_s_*) by IRTs and TCs with air temperature by PRTs. Bias, STDd, RMSE in °C, and results of the linear regression analysis (*y* = *bx* + *c*) ^1^ are given.

Data	*y*	*x*	(*b*, *c*)	*r* ^2^	RMSE	Bias	STDd	*n*
All	*T_s_* (IRT)	*T_a_* (PRT)	1.02, 4.9	0.51	7.86	5.16	5.93	21,004
Daytime			0.97, 8.1	0.35	10.51	7.69	7.16	10,509
Nighttime			0.79, 4.7	0.81	3.63	2.64	2.50	10,495
Day clear sky			1.14, 11	0.33	15.35	12.48	8.95	3532
All			1.06, 4.7	0.63	7.55	5.32	5.35	24,903
Daytime	*Ts* (TC)	*T_a_* (PRT)	1.03, 7.4	0.46	10.29	7.79	6.72	11,447
Nighttime			0.86, 4.5	0.87	3.92	3.22	2.24	13,456
Day clear sky			1.23, 9.5	0.44	15.00	12.33	8.54	3762

^1^ Same as in Table 3.

**Table 6:** Comparison of *T_s_* (IRT) with *T_s_* (LWR) and *T_a_* for the four grassland sites. Bias, STDd, RMSE in °C, and results of the linear regression analysis (*y* = *bx* + *c*) ^1^ between measurements are given.

Site Name	*y*	*x*	Data	(*b*, *c*)	*r* ^2^	RMSE	Bias	STDd	*n*
Audubon	*T_s_* (IRT)	*T_s_* (LWR)	Day	1, 1.4	1	1.53	1.53	0.07	9114
			Night	1.01, 1.4	1	1.45	1.45	0.07	8454
	*T_s_*	*T_a_*	Day	1.28, −0.54	0.8	7.48	4.98	5.57	9114
			Night	1.01, −1.4	0.96	1.98	−1.38	1.42	8454
Brookings	*T_s_* (IRT)	*T_s_* (LWR)	Day	1.04, 1	1	2.2	1.32	1.76	10,928
			Night	0.99, −0.39	1	0.8	−0.38	0.7	6544
	*T_s_*	*T_a_*	Day	1.03, 0.66	0.98	2.35	0.86	2.18	10,928
			Night	0.98, 1.4	0.99	2.04	−1.44	1.44	6544
Canaan Valley	*T_s_* (IRT)	*T_s_* (LWR)	Day	0.97, 0.04	0.99	1.27	−0.25	1.23	9156
			Night	0.96, 0.27	0.98	1.19	0.16	1.17	7588
	*T_s_*	*T_a_*	Day	1.09, 0.73	0.93	3.33	1.57	2.94	9145
			Night	0.97, −1.6	0.97	2.26	−1.72	1.47	7591
Fort Peck	*T_s_* (IRT)	*T_s_* (LWR)	Day	1.01, 0.18	1	0.89	0.36	0.81	9147
			Night	0.98, 0.19	1	0.66	0.18	0.63	7291
	*T_s_*	*T_a_*	Day	1.15, 2.1	0.94	5.75	3.69	4.41	9147
			Night	0.99, −0.37	0.99	1.28	−0.37	1.22	7261
	*T_s_* (LWR)	*T_s_* (LWR)	All	1.03, 0.02	0.99	1.69	0.24	1.68	16,408
	(SEBN)	(SURFRAD)							
	*T_s_* (IRT)	*T_s_* (LWR)	All	1.03, 0.25	0.99	1.97	0.52	1.91	16,408
	(SEBN)	(SURFRAD)							
	*T_s_* (IRT)	*T_s_* (LWR)	All	1.06, 0.46	0.99	2.33	1.05	2.08	9182
	(USCRN)	(SURFRAD)							
	*T_s_* (IRT)	*T_s_* (IRT)	All	0.97, −0.13	0.99	1.44	−0.45	1.37	8523
	(SEBN)	(USCRN)							
	*T_s_* (LWR)	*T_s_* (IRT)	All	0.96, −32	0.99	1.67	−0.77	1.48	8523
	(SEBN)	(USCRN)							

^1^ Same as in Table 3.
